# Detection of Dibutyl Phthalate in Surface Water by Fluorescence Polarization Immunoassay

**DOI:** 10.3390/bios13121005

**Published:** 2023-11-29

**Authors:** Liliya I. Mukhametova, Madina R. Karimova, Olga G. Zharikova, Andrey V. Pirogov, Valentina V. Levkina, Ekaterina S. Chichkanova, Liqiang Liu, Chuanlai Xu, Sergei A. Eremin

**Affiliations:** 1 Faculty of Chemistry, M. V. Lomonosov Moscow State University, Leninskie Gory 1, 119991 Moscow, Russia; liliya106@mail.ru (L.I.M.); olya161197@mail.ru (O.G.Z.); newandry@mail.ru (A.V.P.); levkinavv@my.msu.ru (V.V.L.); chichkanova.ekaterina@mail.ru (E.S.C.); 2 A. N. Bach Institute of Biochemistry, Research Center of Biotechnology, Russian Academy of Sciences, Leninskie Prospect 33, 119071 Moscow, Russia; 3 School of Food Science and Technology, Jiangnan University, 1800 Lihu Road, Wuxi 214122, Chinaxcl@jiangnan.edu.cn (C.X.)

**Keywords:** fluorescence polarization immunoassay, phthalates, dibutyl phthalate, contamination, water safety

## Abstract

Dibutyl phthalate (DBP) is widely used as a plasticizer in the production of polymeric materials to give them flexibility, strength and extensibility. However, due to its negative impact on human health, in particular reproductive functions and fetal development, the content of DBP must be controlled in food and the environment. The present study aims to develop a sensitive, fast and simple fluorescence polarization immunoassay (FPIA) using monoclonal antibodies derived against DBP (MAb-DBP) for its detection in open waters. New conjugates of DBP with various fluorescein derivatives were obtained and characterized: 5-aminomethylfluorescein (AMF) and dichlorotriazinylaminofluorescein (DTAF). The advantages of using the DBP-AMF conjugate in the FPIA method are shown, the kinetics of binding of this chemical with antibodies are studied, the analysis is optimized, and the concentration of monoclonal antibodies is selected for sensitivity analysis—16 nM. The calibration dependence of the fluorescence polarization signal for the detection of DBP was obtained. The observed IC50 (DBP concentration at which a 50% decrease in the fluorescence polarization signal occurs, 40 ng/mL) and the limit of detection (LOD, 7.5 ng/mL) values were improved by a factor of 45 over the previously described FPIA using polyclonal antibodies. This technique was tested by the recovery method, and the high percentage of DBP discovery in water ranged from 85 to 110%. Using the developed method, real water samples from Lake Onega were tested, and a good correlation was shown between the results of the determination of DBP by the FPIA method and GC-MS. Thus, the FPIA method developed in this work can be used to determine DBP in open-water reservoirs.

## 1. Introduction

Environmental pollution is a serious environmental problem. Surface water pollution not only threatens aquatic flora and fauna, but also affects human health [1,2,3,4,5]. Phthalic acid esters (PAE) are widely used as plasticizers in the production of plastics. They have found application in the production of finishing materials, varnishes, paints, perfumes, toys, medical products, disposable tableware, packaging materials and food containers [6,7,8,9,10]. The demand for phthalates has been steadily increasing due to low production costs and the lack of low-cost alternatives. Global production of phthalates has reached approximately 8 million tons per year [11,12]. Dibutyl phthalate (DBP) is one of the most common plasticizers used in the manufacture of plastic products. Usually, DBP is added to polymer from 10% to 60% by weight [9]. Since phthalates are not chemically bound to plastic, DBP is easily released from it and enters aquatic systems through means such as wastewater discharges, urban and agricultural runoff, landfills, and other sources. [5]. Entering the environment, phthalates pollute water [12], soil, air and food [9,13,14]. Recently, there has been growing concern about phthalate contamination of water resources around the world and recent research studies from various countries have reported the prevalence, exposure pathways, toxicity, and impacts of PAE in aquatic ecosystems and humans, as summarized in this recent review [12]. It has been shown that DBP is able to accumulate in the human body, which leads to general hormonal failure, negatively affects the functioning of the kidneys and liver, causes cancer [10,15], destroys the endocrine system and reduces fertility [16]. Phthalates are particularly harmful to the health of children and adolescents, which has been repeatedly demonstrated in clinical studies [17,18] and hospitalized patients [19]. Since DBP poses a great threat to human health and also poses a danger to the environment, it was classified as a hazardous substance subject to priority control [20].

Permissible levels of phthalates in water and food are regulated [21]. In addition, due to the negative impact of PAE on the human endocrine system, environmental quality standards were established based on average annual concentrations in freshwater environments; thus, the maximum permissible concentration (MPC) of DBP in open water and groundwater in the EU countries is 10 µg/L, the maximum acceptable concentration for aquatic ecosystems is 35 μg/L and the maximum acceptable concentration for aquatic ecosystems of 0.43 mg/L [22,23]. The maximum acceptable concentration of dibutyl phthalate in water established in Russia is 0.2 mg/L [24], while the US Environmental Protection Agency (USEPA) maximum contaminant levels (MCL) for DBP are higher—0.45 mg/L [25].

Thus, the development of sensitive and selective analytical methods for the detection of PAE, in particular DBP, is of great importance for monitoring their content in the waters of open reservoirs. In particular, there is a need for methods that allow these studies to be carried out outside of the laboratory. The development of new, rapid and sensitive methods for point-of-need determination of environmental pollutants is an increasingly promising direction. The emergence of new compact devices expands the possibilities for their implementation [26,27,28].

Currently, the analysis of PAE is mainly carried out by chromatographic methods, for example, high-performance liquid chromatography (HPLC) [29], ultra HPLS using a sustainable natural deep eutectic solvent-based analytical methodology [30] or gas chromatography-mass spectrometry (GC-MS) [8,9,31,32,33,34,35,36]. These analytical methods allow accurate and sensitive quantitative analysis; however, they require expensive instrumentation, highly skilled personnel and complex sample processing that cannot meet the requirements for rapid detection of toxicants outside the laboratory. Immunosensoric determination for DBP using carbon quantum dots was conducted to overcome some of the abovementioned limitations, but these techniques are not applicable for on-site rapid testing [37,38]. Therefore, the development of sensitive and selective analytical methods for rapid and accurate detection of PAE at the point of need is necessary.

Immunoassay methods have great advantages over chromatographic methods due to their relatively low cost and implementation simplicity. For DBP determination, there are commonly used immunological techniques such as enzyme-linked immunosorbent assay (ELISA) [39,40,41], immunochromatographic (ICA) [42] and fluorescence polarization immunoassay (FPIA) [43,44].

Among the immune methods of analysis, ELISA is the most widely used in areas such as the diagnosis of diseases [45], food safety [46] and environmental monitoring [47]. It has obvious advantages, including low cost, good specificity, automation and high performance. However, traditional colorimetric ELISA is often accompanied by false positive responses and has a long analysis time [48]. The ICA method of analysis is fast and can be performed directly on site, but this method can give unreliable results in the presence of various phthalates in the sample and is sensitive to changes in the pH and composition of the sample. In addition, ICA generally provides only a qualitative or semiquantitative assessment of the content of the analyte. FPIA has several advantages over the abovementioned immune methods: it is a homogeneous method, does not require separation of the free form from the bound one, is carried out quickly, provides the results in a few minutes and can be carried out directly at the sampling site The principle of FPIA is based on the phenomenon of fluorescence polarization. When stimulated with plane-polarized light, a small free fluorescent molecule, typically <1.5 kDa (tracer), primarily emits unpolarized light. This is due to the fact that a small free molecule, during the time between excitation and emission rapidly rotates in the solution and, accordingly, emits light in different polarization planes (low FP signal). Conversely, if a small fluorescent molecule is bound to a larger one (usually >10 kDa), its increased molecular volume slows its rotation and results in the emission of light predominantly polarized in the same plane as the excitation source (high FP signal). An increase in the concentration of the free antigen causes competition for binding with antibodies and a decrease in the fluorescence polarization signal due to the transition of the indicator from the state bound to the antibody to the free state, depending on the concentration of the analyte [49]. This effect is used in FPIA to determine the concentration of the target antigen in the sample. The developed FPIA formats for the determination of DBP using immunoreagents of the conjugate of the amino derivative of fluorescein with succinate-dibutyl phthalate (DBP-EDF) and polyclonal antibodies obtained against DBP have a fairly high detection limit (350 ng/mL) [43]. In another FPIA assay, instead of antibodies, the recombinant receptor protein mPPARα-LBD was used as a recognition element and fluorescently labeled nonanoic acid (C4-BODIPY-C9) [44]. This fluorescence polarization assay can simultaneously detect multiple PAE with different sensitivities and specificities. Given that real samples may contain more than one PAE, the proposed method is favorable for determining the total amount of PAE.

However, one of the most toxic and commonly used phthalic acid esters is DBP. Its content is currently strictly controlled in water bodies. Therefore, to meet the requirements for the detection of DBP, a more sensitive and accurate method of investigation is needed. The above literature review showed that homogeneous FPIA allows quantitative, rapid and specific determination of low-molecular pollutants in various objects both in laboratories and directly at the points of need, thanks to the availability of portable analyzers. However, previously described FPIA techniques for DBP have not fully realized these advantages due to poor sensitivity [43] or low selectivity [44]. For the development of highly sensitive FPIA, the quality of immunoreagents, both antibodies and tracers, plays a decisive role. The use of highly specific monoclonal antibodies instead of polyclonal rabbit serum and the production of a new purified tracer allows developing a highly selective and sensitive FPIA with a significant lowering of its detection limit.

## 2. Materials and Methods

### 2.1. Reagents and Materials

Dibutyl phthalate (DBP), dimethyl phthalate (DMP), diethyl phthalate (DEP), diisobutyl phthalate (DisoBP), diethylhexyl phthalate (DEHP), dioctyl phthalate (DOP), butyl benzyl phthalate (BBP), monomethyl phthalate (MMP), monobenzyl phthalate (MBP), monohexyl phthalate (MHP), 5-(Aminomethyl)Fluorescein Hydrochloride (AMF), *N*-hydroxysuccinimide (NHS), *N*,*N*’-dicyclohexylcarbodiimide (DCC) were acquired from Sigma Aldrich Corporation (Saint Louis, MO, USA). Dichlorotriazinylaminofluorescein (DTAF), sodium azide were purchased from Serva (Heidelberg, Germany). Triethylamine was acquired from Merck (Darmstadt, Germany). Chloroform, methanol, dimethylformamide (DMF) (special purity grades) were purchased from Khimmed (Moscow, Russia). The 50 mM borate buffer solution (pH 8.5) containing 0.1% NaN3 was used. Stock solutions of phthalates were prepared at a concentration of 10 mg/mL in methanol, from which standard solutions were prepared in 10% methanol.

The DBP-AMF conjugate was purified by thin-layer chromatography on precoated TLC sheets ALUGRAM^®^Xtra SIL G/UV_254_ (Macherey-Nagel, Düren, Germany). The fluorescence polarization assays were conducted on a portable Sentry-200 fluorimeter (Ellie LLC, Germantown, WI, USA) in a tube made from borosilicate glass, size 10 × 75 mm. Light source LED, detector—photomultiplier tube, λex = 485 and λem = 535 nm. The obtained data were processed using the Sigma Plot 11 software package (Systat Software Inc., Palo Alto, CA, USA).

### 2.2. Synthesis of the Fluorescein Labeled Antigen (Tracer)

The synthesis of conjugates of dibutyl phthalate with DTAF was carried out by dissolving 5 mg of 4-amino-dibutyl phthalate in 500 μL of methanol with 20 μL of trimethylamine and adding 10 mg of DTAF. The mixture was incubated for 24 h in the dark.

The preparation of the DBP-AMF conjugate was carried out in two stages: (1) Activation of the carboxyl group of DBP-Su: the molar ratio of DBP-Su: DCC: NHS during the synthesis was 1:2:2. DCC (6.5 mg) and NHS (3.8 mg) were dissolved in 200 µL of DMF. DBP-Su (5 mg) was then added and the solution was incubated for 18 h at room temperature. The reaction mixture was centrifuged at 10,000× *g* for 3 min and the supernatant was collected. (2) The addition of fluorescent dye: AMF (10 mg) and 20 μL of trimethylamine were added to the supernatant and incubated for 24 h in the dark. The synthesis of the tracers was conducted in three independent experiments.

#### Purification of the Tracer

DBP-AMF and DBP-DTAF conjugates were purified from impurities by thin-layer chromatography using methanol: chloroform as an eluent in a ratio of 1:4 (*v*/*v*). The main yellow fluorescent band (Rf = 0.9) was collected from the chromatographic plate and extracted with 1 mL of methanol (Figure S1). The purification of tracers was conducted sequentially in two stages. The concentration of DBP-AMF and DBP-DTAF conjugates was determined spectrophotometrically, by absorbance at 492 nm in 50 mM carbonate buffer solution, pH 9.0, using a fluorescein molar extinction coefficient of 8.78 × 10^4^ (L/mol*cm) [50]. The specificity of the obtained tracers was determined by studying their binding to specific monoclonal antibodies obtained against DBP. The DBP-AMF structure was checked by high-resolution tandem mass spectrometry coupled with high-performance liquid chromatography, UV spectroscopy (Figures S3–S5).

### 2.3. Producing Monoclonal Antibody

Monoclonal antibodies against DBP were obtained using BALB/c mice (1.5–2 months) by hybridomic technology with antigen DBP-BSA as described [41]. Animal studies were conducted in accordance with the EU Directive 2010/63/EU and are authorized by the Ethics Committee of the Biotechnology Research Center (Protocol N22-D of 12 February 2020).

### 2.4. FPIA for DBP

#### 2.4.1. Kinetics of MAbs—Tracer Interaction

MAb-DBP (16 or 10 nM) and tracer DBP-AMF (5 nM) in 50 mM borate buffer, pH 8.5, were mixed in a test tube (500 µL both). The mP values were measured at 25 °C for 2 h. The kinetic curves were approximated by an exponential association equation (choosing the most appropriate values of *y*0, *a* and *b*):

(1) $$mP=y0+a*(1-\exp\left(-b*t\right))$$

where mP denotes the varied FP signal (the *y* axis value), *y*0 denotes the FP signal of free DBP-AMF, *t* is time (the *x* axis value), and *a* and b are parameters termed the observed kinetics of the signal growth.

#### 2.4.2. Competitive FPIA

The standard solution of 500 μL of various concentrations of DBP in 5% methanol (0–10,000 ng/mL) and 500 μL of a DBP-AMF conjugate solution with the selected optimal concentration 5 nM were added to glass tubes. Then, 50 µL of a solution of MAb-DBP antibodies with a concentration of 24 or 48 µg/mL was added, and after 60 min of incubation at 25 °C, measurements were made on a Sentry-200 portable fluorimeter (Ellie LLC, USA). Each measurement was carried out in triplicate. Based on the measurement results, the dependence of the fluorescence polarization on the DBP concentration (calibration curve) was obtained using a semi-logarithmic scale for the DBP concentration, which was approximated by a four-parameter sigmoid function (2) using Sigma Plot 11 software (Systat Software Inc., Palo Alto, CA, USA).

(2) $$mP={mP}_{0}+\frac{{mP}_{max}-{mP}_{0}}{{1+(\frac{\left[DBP\right]}{IC50})}^{Hillslope}}$$

where mP is the measured fluorescence polarization signal, mP_0_ is the free tracer polarization, mP_max_ is the fluorescence polarization signal for the MAb-DBP*DBP-AMF complex in the absence of free DBP, and IC50 is the DBP concentration at which a 50% change in FP occurs.

### 2.5. FPIA Specificity

The specificity of FPIA has been studied under optimal experiment conditions. Crossreactivity (CR) was estimated using IC10 values (concentration at which a 10% decrease in the fluorescence polarization signal occurs). Competitive FPIA was conducted using other phthalic acid esters, DMP, DEP, DisoBP, DOP, DEHP, and BBP, and their metabolites monophthalates, MMP, MBP, and MHP, in the concentration range 1–1000 ng/mL.

### 2.6. Preparation of Water Samples and Recovery Test

All water samples were obtained from Lake Onega and stored at 4 °C. Water samples that did not contain DBP by GC-MS were used to check FPIA by the recovery test. Before FPIA, these water samples were spiked with DBP and analyzed without subsequent sample preparation. All experiments were duplicated.

### 2.7. Determination of DBP in Water Samples by GC-MS

Water samples were prepared for analysis in glass flasks with ground-in lids. Phthalates were extracted with n-hexane (10 mL sample + 1 mL n-hexane). The determination was carried out by gas chromatography-mass spectrometry in the mode of registration of isolated ions [51,52]. Briefly, the column HP-5MS (30 m × 250 microns × 0.25 microns) (“Agilent Technologies”, Santa Clara, CA, USA) was used, and the mobile phase was helium and the eluent flow rate was 1 mL/min. The volume of the injected sample is 1 µL. Temperature gradient was from 40 to 130 °C at a speed of 50 °C/min, from 130 to 250 °C at a speed of 5 °C/min, and from 250 to 300 °C at a speed of 10 °C/min. The source temperature was 230 °C. Mass spectrometric detection was performed on the following ion: *m*/*z* 149 [51,52]. An auxiliary *m*/*z* 223 peak was used to confirm the detection of DBP [53].

The study was carried out using the equipment of the Central Collective Use Center of Moscow State University “Technologies for Obtaining New Nanostructured Materials and Their Comprehensive Study”.

## 3. Results and Discussion

### 3.1. Obtaining and Characterization of the Specific Reagents

**Figure 1:**
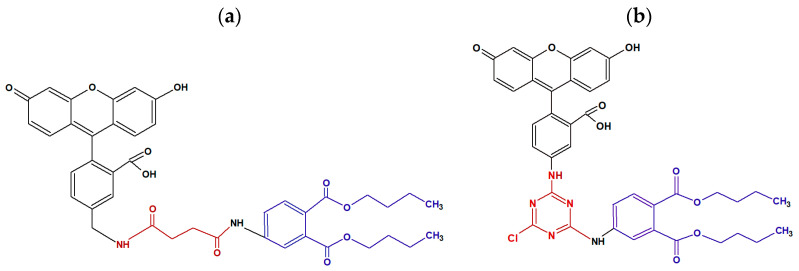
Structures of DBP–AMF (**a**) and DBP–DTAF (**b**) tracers. The hapten (DBP) is marked with blue, and the fluorescein is marked with black and spacer between them is red.

For the development of a highly sensitive FPIA assay, the quality of the immunoreagents plays an important role [54,55]. A special role is assigned to the conjugate of a low molecular weight analyte with a fluorescent label—a tracer [56]. It is important that the synthesized tracer effectively binds to antibodies and represents a highly purified preparation. In the previous study [43], a fluorescently labeled conjugate with a carboxylated derivative of DBP (DBP-Su) with ethylenediamine fluoresceinthiocarbamate (EDF) was synthesized. However, the DBP detection limit using this tracer was quite high and amounted to 350 ng/mL. In this work, to obtain DBP tracers with fluorescein derivatives, two haptens were used, 4-amino-DBP and 4-succinate-DBP, which have different functional groups, NH_2_– and –COOH, respectively. Therefore, to obtain tracers, DTAF and 5-AMF were used, which react with these haptens [54,55]. Note that the structures of DBP-AMF and DBP-DTAF (Figure 1) tracers differ in such parameters as length and rigidity of the spacer between fluorescein and DBP. The FPIA tracer (conjugate of a low molecular weight analyte with a fluorescent label) plays an important role in the sensitivity, accuracy and stability of analysis. The tracer contains three groups: ligand, fluorescent label and the linker between the ligand and the fluorophore. The length of the linker between the hapten and fluorescein affects the sensitivity of FPIA [57,58]. For example, short linkers between the fluorophore and the antigen can minimize the “propeller effect”, and a too short linker can affect the binding affinity of the tracer [57]. However, the conjugation of a fluorescent label with a longer spacer can lead to a more sensitive FPIA analysis [58].

**Figure 2:**
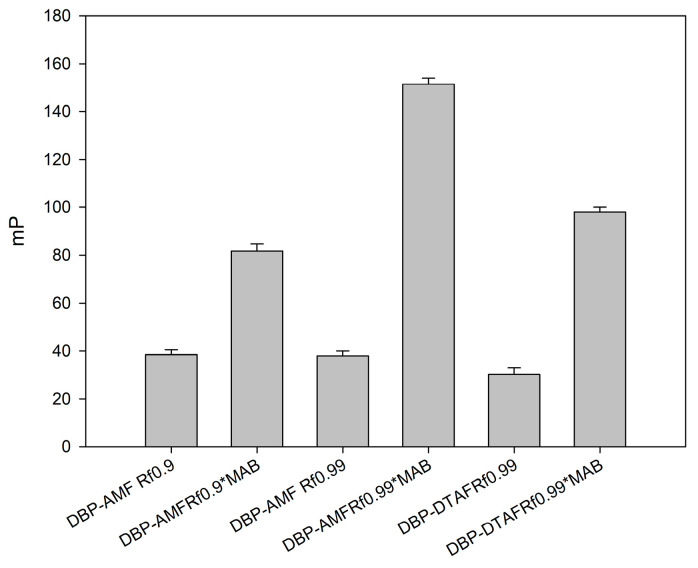
Dependence of the fluorescence polarization signal on the degree of tracer purification. Finish tracer concentrations were 2.5 nM, and the concentration of antibodies was 10 nM, 25 °C, pH 8.5 (n = 3).

For the development of FPIA, the DBP-AMF and DBP-DTAF tracers were synthesized (the structures are shown in Figure 1). The obtained conjugates were purified by thin-layer chromatography (TLC) and main fractions with a retention factor (Rf 0.9) were studied. To check the resulting conjugates, working solutions of each fraction were prepared, and dilutions of stock tracer solutions were selected so that their fluorescence intensity exceeded the background signal of a pure buffer by a factor of 20. The concentration of tracers working solutions was 5 nM. At a given concentration, the tracers gave the optimal signal/noise ratio and, consequently, a stable value of the fluorescence polarization. The main characteristic of tracers is their binding to specific antibodies. Therefore, we undertook a study of the binding of our tracers to monoclonal antibodies obtained against DBP. The resulting monoclonal antibodies against DBP (MAb-DBP) were added to the working solutions of the tracers DBP-AMF and DBP-DTAF, and the FP signal was measured (Figure 2). Both tracers DBP-AMF and DBP-DTAF were shown to bind to MAb-DBP. It is known that the development of highly sensitive FPIA requires a high quality of immunoreagents [56]. Therefore, we undertook additional purification of tracers by TLC; the obtained conjugates DBP-AMF Rf 0.99 and DBP-DTAF Rf 0.99 were also tested for binding to the monoclonal antibody MAb-DBP. The results are shown in Figure 2. The tracer fluorescence polarization signal after repeated purification increased markedly when bound to antibodies.

The synthesis of tracers was repeated in three independent experiments and their functional properties (antibody binding) were studied. We obtained reproducible results on their functional properties (antibody binding) (Figure S2). The tracer working solution was stable for more than 8 h in 50 mM borate buffer, pH 8.5. During this time, the binding ability of the tracers to antibodies was maintained. However, it can be seen from the presented data that the increase in the FP signal upon binding to antibodies was higher for the DBP-AMF tracer than for DBP-DTAF, which is probably due to steric hindrances that may arise during the interaction of antibodies with DBP-DTAF.

The structure of the DBP-AMF tracer was verified spectrophotometrically and chromatographically with MS detection (Figures S3–S5).

### 3.2. Studying of Binding Kinetic DBP-AMF and DBP-DTAF with Monoclonal Antibody

**Figure 3:**
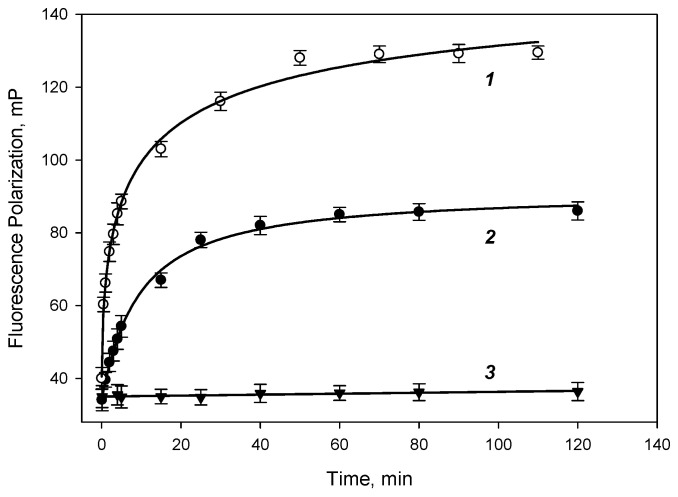
Binding kinetics of DBP-AMF (2.5 nM) with monoclonal antibody MAb-DBP 16 (curve 1), 10 nM (curve 2) and antibody MAb-antiPG 10 nM (curve 3) in a 50 mM borate buffer pH 8.5, 25 °C, (n = 3). The chosen parameters of approximation for curves 1–3 are given in the Supplementary Materials.

The antibody binding kinetics to DBP-AMF and DBP-DTAF tracers were studied by measuring the fluorescence polarization signal; 500 µL of borate buffer, 500 µL of the working solution of the DBP-AMF tracer and 0.05 mL of antibody solution were mixed in a test tube with a known concentration. The tube was placed in a Sentry-200 portable fluorimeter and the kinetics of binding of the DBP-AMF tracer to antibodies was studied. Figure 3 shows the kinetic curves of the binding of the tracer DBP-AMF and DBP-MAb at two concentrations of 16 and 10 nM, respectively. Note that as the concentration of antibodies increases, the association rate increases (the slope on the binding curve increases). Complete binding was achieved within 50–60 min of incubation at room temperature. The kinetic curves were approximated by exponential association (Equation (1)). The binding of the DBP-DTAF tracer took place almost immediately when the solutions were mixed, and the equilibrium was established within 1–2 min. Then, while studying the binding of antibodies to DBP-AMF, we measured the fluorescence polarization signal after 60 min of incubation, and for the DBP-DTAF tracer after 2 min. We also studied the stability of the fluorescence polarization signal for 4 h and showed that the change in the FP signal during this time did not exceed 1–2 units. In addition, the binding specificity of the tracers DBP-AMF and DBP-DTAF was tested with a monoclonal antibody that was obtained against progesterone: MAb-antiPG. The value of the polarization of the tracer fluorescence practically did not change (Figure 3), which indicates a highly specific binding of this tracer only with antibodies obtained against DBP (Figure 3 (curve 3)).

### 3.3. Optimization FPIA for DBP Determination and Studying of Method Crossreactivity

The combination of antibody/tracer has a significant impact on the specificity and sensitivity of competitive FPIA [43]. To optimize the method, we also selected the concentration of antibodies. The competitive FPIA format is based on the fact that free and fluorescently bound antigens compete for a limited number of antibody binding sites [59,60]. The antibody concentration is selected during the experiment so that the detection limit is minimal, and the change in the fluorescence polarization signal (ΔmP) in the absence of antigen and its maximum amount is sufficient to accurately determine the analyte concentration.

**Figure 4:**
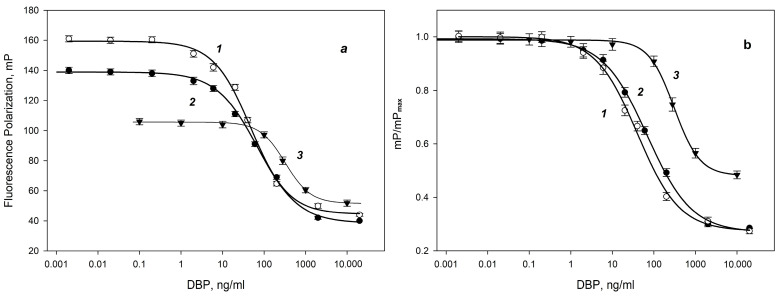
Dependence of the fluorescence polarization signal in the competitive FPIA format at different concentrations of MAb-DBP antibodies (**a**) and normalized curves (**b**) in the reaction mixture of 16 (○-curve 1) and 8 nM (●-curve 2) for DBP-AMF 2.5 nM and 16 nM MAb-DBP and (▼-curve 3) DBP-DTAF 2.5 nM (25 °C, pH 8.5).

For the DBP-AMF tracer, two calibration dependences of the change in the fluorescence polarization signal on the DBP concentration were obtained at different concentrations of antibodies (8 and 16 nM) and their analytical characteristics were analyzed (Figure 4) [59,60]. Immunoassays generally do not follow a linear dose–response relationship. FPIA often produces a sigmoidal curve, as in Figure 4. The four-parameter logistic curve is used to analyze bioassays such as FPIA and ELISA (Equation (2)). The detection limit (LOD) was determined at the initial section of the graph (Figure 4 (curve 1)) corresponding to the DBP concentration with fluorescence polarization equal to mP = mPmax-3S, where mPmax is the fluorescence polarization signal of standard solution without DBP and S is the standard deviation. The sensitivity FPIA was determined as IC50 analysis (the concentration causing a 50% decrease in the signal). Using antibodies at concentrations of 16 and 8 nM, the DBP LOD was 7.5 ± 0.2 and 10.0 ± 0.2 ng/mL, and IC50 was 40 ± 2 and 70 ± 2 ng/mL, respectively. As can be seen from Figure 4b, the detection limit of DBP and IC50 at an antibody concentration of 16 nM is lower than at a concentration of 8 nM. Increasing the antibody concentration to 20 nM resulted in a decrease in LOD to 10 ng/mL. Thus, when using an antibody concentration of 16 nM, we obtained a more sensitive analysis. For the DBP-DTAF tracer, at an antibody concentration of 16 nM, a higher detection limit (93 ± 1 ng/mL) was obtained than for the DBP-AMF tracer. The structure of the best tracer DBP-AMF was confirmed by high-resolution tandem mass spectrometry coupled with high-performance liquid chromatography (see Figure S1). Additional experiments confirming the composition of the tracer are presented in the Supplementary Materials. As can be seen from the presented data, the tracer with DBP-AMF with a more flexible spacer reacted slowly (about 1 h) with the monoclonal antibody against DBP than the tracer with a more rigid spacer DBP-DTAF (binding time 1–2 min). It was shown that by using the DBP-AMP tracer, it was possible to obtain a more sensitive analysis. Probably, the longer binding time of the DBP-AMP tracer with antibodies is the factor causing a lower detection limit for DBP in the competitive FPIA format with the DBP-AMP tracer.

**Figure 5:**
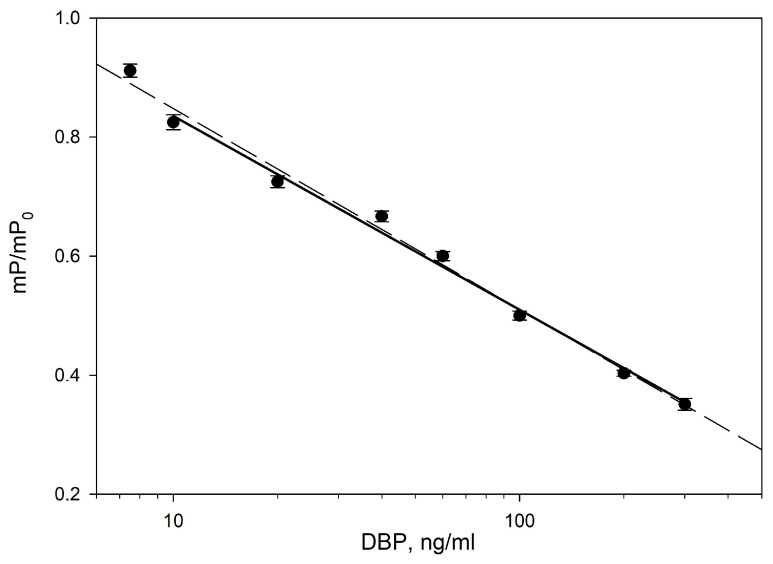
The detection range of the normalized calibration dependence of the DBP determination using a pair of MAb-DBP/DBP-AMF immunoreagents has final concentrations of 16/2.5 nM, respectively. 25 °C. pH 8.5. Solid line corresponds IC20-IC80 (20 and 80% FP signal variation range); the dashed line includes LOD in the detection range. mP/mPmax = (1.14 ± 0.02) − (0.32 ± 0.01)*[DBP], r2 = 0.993; where mP is the measured fluorescence polarization signal and mPmax is the fluorescence polarization signal for the MAb-DBP*DBP-AMF complex in the absence of free DBP.

Thus, the optimal pair of immunoreagents MAb-DBP/DBP-AMF is at concentrations of 16 nM/2.5 nM (Figure 4b curve 1). The calibration dependence is linearized in semi-logarithmic coordinates and is presented in Figure 5. This curve was evaluated in accordance with the recommendations [61] and the detection range was 10–300 ng/mL (Figure 5 (solid line)), the selected range being IC20-IC80 (20 and 80% FP signal variation range). This detection range reflects the most accurate measurements with an error of less than 10%. This calibration dependence (Figure 5) was used to check the FPIA method by the recovery test and analysis of real water samples.

We have studied the crossreactivity (CR) of other phthalates structurally similar to DBP. For this objective, the following phthalic acid esters and their metabolites monophthalates were tested: DMP, DEP, DisoBP, DOP, DEHP, BBP, MMP, MBP, and MHP in the concentration range 1–1000 ng/mL. However, for all checked di- and monoesters, the degree of signal inhibition was low and did not reach even 10%. So, the equation for % CR based on IC50 cannot be applied, and the crossreactivities were estimated using IC10 values. Comparing IC10 for the calibration curve and signals for the maximal tested concentration of cross-reactants, we can conclude that % CR is less than 1% for all tested phthalic acid esters and their metabolites, which indicates a highly specific analysis for DBP.

### 3.4. Determination DBP in Real Samples

Pollution of the waters of open reservoirs with phthalates is a potential danger not only for the flora and fauna of reservoirs, but also for humans [1,2,3]. Therefore, it is necessary to control the levels of phthalic acid esters in surface waters of open reservoirs [12,35,36]. To determine the level of phthalate contamination of Lake Onega, 16 water samples were collected. These samples were initially tested by GC-MS [50,52]. After analysis, we selected two samples that did not contain phthalates (#1 and #2). The spiked samples containing exact concentrations of DBP were prepared (finish concentration 30 and 90 ng/mL). These spiked samples were stored at room temperature overnight and analyzed using the FPIA. Table 1 demonstrates the high recovery percentage (86–110%). This result confirms that this method can be used to determine DBP in real water samples.

With our assistance for calibration, other water samples obtained from Lake Onega were also tested, in which the content of DBP was confirmed by GC-MS (Table 2). A notable feature observed in the mass spectra of phthalates is the presence of a common fragment ion of protonated phthalate anhydride with a mass-to-charge ratio of *m*/*z* 149. This mass peak is characteristic of most phthalates with longer alkyl substituents and was used to identify DBP [52]. An auxiliary *m*/*z* 223 peak was used to confirm the detection of DBP (Figure S6) [54]. The majority of samples we analyzed contained DBP below the detection limit of FPIA, or at the sensitivity limit of our method (Table 2). The correlation between the results of DBP determination in real water samples by GC-MS and FPIA is presented in Figure S6. The water samples with FPIA results with lower LOD were excluded from the plot. The Pearson coefficient was calculated using the Sigma Plot 11 software package. This coefficient is 0.975 (Figure S7), which corresponds to a good correlation between the results obtained by FPIA and GC-MS. This result further confirmed the reliability of the FPIA for the detection of DBP.

As we discussed, there are few immunoassay methods for DBP detection described in the literature. Table 3 summarizes the data on the sensitivity and types of matrices tested by the proposed analyses. As presented in the table below, immunochemical methods of analysis have good sensitivity and a low detection limit. In the literature, FPIA methods for determining DBP have been described and, as can be seen from the data presented, the use of monoclonal antibodies has reduced the detection limit of DBP compared to the previously described method [43] by 45 times.

## 4. Conclusions

In this work, a sensitive FPIA method for the determination of DBP was developed using a new pair of immunoreagents, DBP-AMF and monoclonal antibodies MAb-DBP. It has been shown that the use of monoclonal antibodies and the production of a highly purified tracer makes it possible to develop a more sensitive and specific FPIA. The developed method makes it possible to determine the specific content of DBP in water samples, since the crossreactivity of other phthalates and their metabolites is less than 1%. The obtained analytical characteristics of the method allow determining the content of DBP in the waters of open reservoirs, and the detection limit of phthalate by the FPIA method (7.5 ng/mL), which is over 20 times lower than the maximum allowable concentration of dibutyl phthalate in water established in Russia (200 ng/mL). In this work, the FPIA method was used to test water samples obtained from Lake Onega, which were previously verified by GC-MS, and showed that the content of phthalates in real water samples is lower than the MPC established in Russia. A comparison of the results of determining DBP by the two methods showed good convergence results. The FPIA method, due to its sensitivity and ease of analysis, can be used to determine the content of phthalates in real samples. In addition, the use of a portable Sentry-200 device allows analysis to be carried out directly at the sampling place.

## Figures and Tables

**Table 1:** Recoveries of DBP from environmental water (n = 3).

Water Sample	Added DBP Concentration, ng/mL	Detected DBP Concentration, ng/mL	Recovery, %
1	30	26 ± 1	86 ± 4
	90	85 ± 3	94 ± 4
2	30	34 ± 2	113 ± 6
	90	98 ± 2	109 ± 2

**Table 2:** Detection of DBP from environmental water by FPIA and GC-MS (n = 3).

Water Sample	GC-MS, ng/mL	FPIA, ng/mL
3	10	ND
4	10.6	10.5
5	10.6	10.0
6	6.0	ND
7	7.5	ND
8	7.3	ND
9	10.5	10.0
10	11.2	10.0
11	7.1	ND
12	5.3	ND
13	16.5	12.5
14	18.1	14.2
15	14.0	11.0
16	8.0	ND

**Table 3:** Studies on the detection of DBP by different immunoassays.

Antibody	Assay Format	Sample	LOD ng/mL	Range ng/mL	Reference
Polyclonal	icELISA	White wine	64.5	64.5–1606.2	[38]
Polyclonal	BA-ELISA	Drinking water	5	0.02–8.97	[39]
Monoclonal	ELISA	Baverange	3.6		[40]
Monoclonal	ICA	Water	33.4	42.4–1500	[42]
Polyclonal	FPIA	Water	350	500–7500	[43]
Receptor	FPIA	Spirite	170	170–8700	[44]
Monoclonal	FPIA	Water	7.5	12–300	This work

## Data Availability

The original contributions presented in the study are included in the article, further inquiries can be directed to the corresponding author.

## Supplementary Materials

The following supporting information can be downloaded at: https://www.mdpi.com/article/10.3390/bios13121005/s1. Figure S1: The results of TLC for DBP-AMF (a,b) and DBP-DTAF (c,d). (a,c)—fist purification, (b,d)—second purification of tracers. (Red arrow represents the band that binds to the antibody); Figure S2: Studying binding properties of DBP-AMF obtained in three independent experiments (1)–(3) with MAb-DBP; Figure S3: Chromatogram of the DBP-AMF sample a) by total ion current and b) by isolated ion of the target compound; Figure S4: Mass spectrum of DBP-AMF sample by the isolated ion of the target compound; Figure S5: UV-spectra pure FITC and tracer DBP-AMF; Figure S6: (a) Fragment of a chromatogram of a water extract containing DBP. (b) Experimental mass spectrum of the peak with a retention time of 11.93 min; Figure S7: Correlation results determination DBP in real water samples by GC-MS and FPIA.
