# Supplementary material for: Detection of Dibutyl Phthalate in Surface Water by Fluorescence Polarization Immunoassay
Source: Biosensors (Basel). 2023 Nov 29;13(12):1005. doi: 10.3390/bios13121005 (PMC10741632; doi:10.3390/bios13121005)
Supplement: Supplementary file 1 [file biosensors-13-01005-s001.zip › biosensors-2672938-supplementary.pdf]

biosensors

MDPI

Article

Detection of Dibutyl Phthalate in Surface Water by Fluorescence Polarization Immunoassay

Liliya I. Mukhametova [1,2], Madina R. Karimova [1], Olga G. Zharikova [1], Andrey V. Pirogov [1], Valentina V. Levkina [1], Ekaterina S. Chichkanova [1], Liqiang Liu [3], Chuanlai Xu [3], Sergei A. Eremin [1,2]*

[1] Faculty of Chemistry, M. V. Lomonosov Moscow State University, Leninskie Gory 1, 119991 Moscow, Russia
[2] A. N. Bach Institute of Biochemistry, Research Center of Biotechnology, Russian Academy of Sciences, Leninsky Prospect 33, 119071 Moscow, Russia
[3] School of Food Science and Technology, Jiangnan University, 1800 Lihu Road, Wuxi, Jiangsu Province, 214122 China

* Correspondence: saeremin@gmail.com; Tel.: +7 916 5127654

Citation: Mukhametova, L.I.; Karimova, M.R.; Zharikova, O.G.; Pirogov, A.V.; Levkina, V.V.; Chichkanova, E.S.; Liu, L.; Xu, C.; Eremin, S.A. Detection of Dibutyl Phthalate in Surface Water by Fluorescence Polarization Immunoassay. *Biosensors* **2023**, *13*, 1005. https://doi.org/10.3390/bios13121005

Received: 19 October 2023
Revised: 19 November 2023
Accepted: 26 November 2023
Published: 29 November 2023

Copyright: © 2023 by the authors. Licensee MDPI, Basel, Switzerland. This article is an open access article distributed under the terms and conditions of the Creative Commons Attribution (CC BY) license (https://creativecommons.org/licenses/by/4.0/).

1. *Purification of obtained conjugates DBP-AMF and DBP-DTAF*

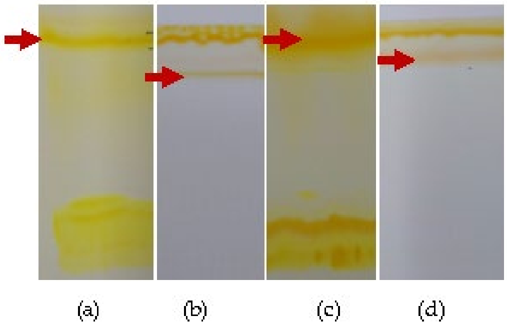

Figure S1. The results of TLC for DBP-AMF (a-b) and DBP-DTAF (c-d). (a) and (c) – fist purification, (b) and (d) – second purification of tracers. (Red arrow represents the band that binds to the antibody).

As discussed in the text of the article, the tracers were cleaned using the TLC method twice. As can be seen in the first chromatograms (a) and (c), a large number of impurities present, but the second purification (b) and (d) completely eliminated them and practically only the target bands presented on the plate.

2. *Functional and structural characteristics DBP-AMF*

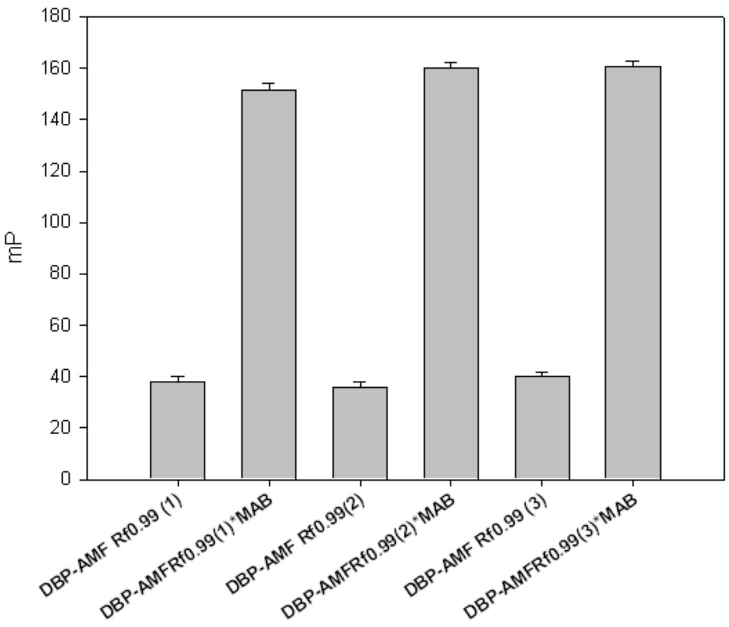

Figure S2. Studying binding properties of DBP-AMF obtained in three independent experiments (1)-(3) with MAb-DBP.

Where (1) – (3) numbers of three experiments on tracer synthesis. We repeated the synthesis and purification of DBP-AMF three times and compared its binding to specific monoclonal antibody Mab-DBP and demonstrated, that all three products bound to antibodies equally effectively.

Structure of DBP was confirmed by mass spectrum. Initially DBP-AMF sample the chromatogram was conducted by total ion and isolated ion of the target compound (Fig.S3). After that mass spectrum of DBP-AMF sample by the isolated ion of the target compound was obtained (Fig.S4).

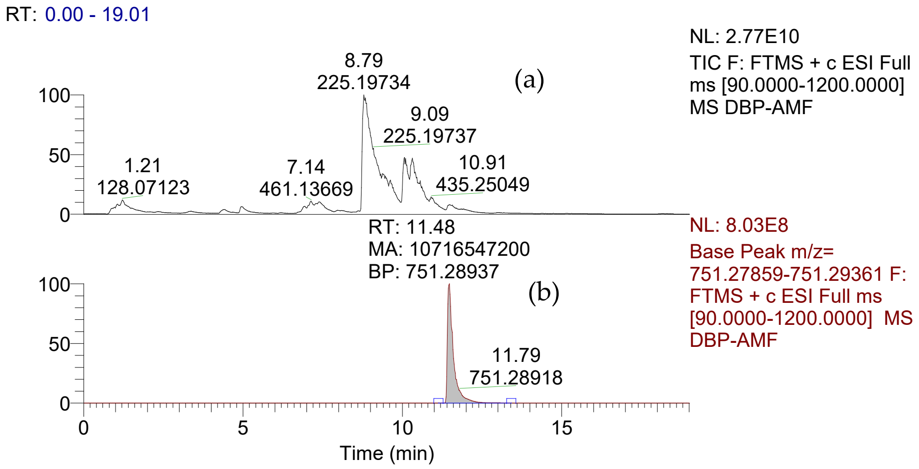

Figure S3. Chromatogram of the DBP-AMF sample a) by total ion current and b) by isolated ion of the target compound.

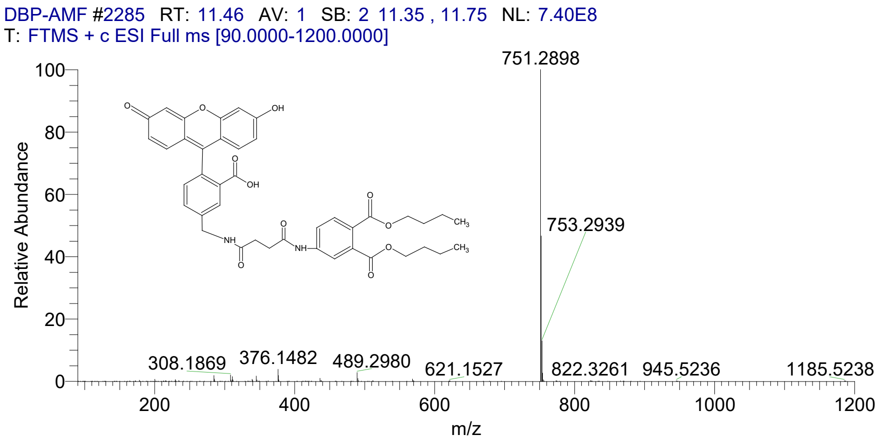

Figure S4. Mass spectrum of DBP-AMF sample by the isolated ion of the target compound.

Mass spectra were obtained using a Q-Exactive tandem mass spectrometer coupled to an Ultimate 3000 high performance liquid chromatograph; samples were ionized by electrospray in a HESI-II ionization source (Thermo Scientific, Waltham, MA USA). Mass spectrum of the first order, the mode of registration of positively charged ions. The first-order mass spectra in the positive ion detection mode showed peaks corresponding to [M+H]+ (m/z 751.2861).

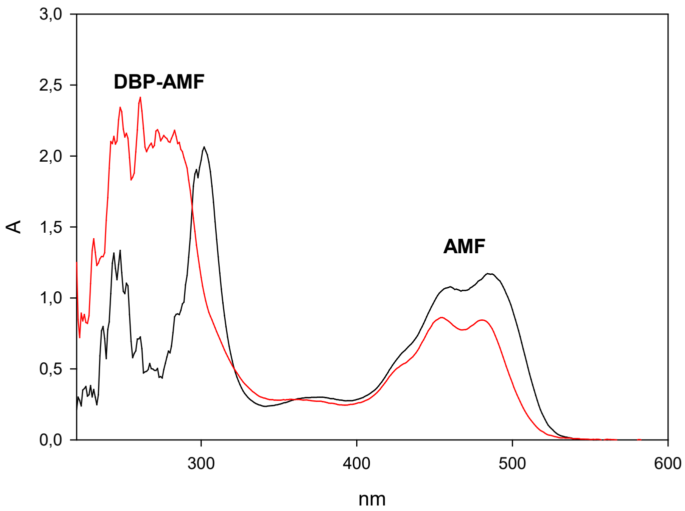

Figure S5. UV-spectra pure FITC and tracer DBP-AMF.

UV-spectra pure AMF and tracer DBP-AMF were studied (**NanoDrop 2000 UV Visible Spectrophotometer (Fisher Scientific, USA))**. The absorption maxima of DBP and FITC observed at 278 and 495 nm, respectively. Figure S3 demonstrates the absorption peaks for DBP and AMP, which indicates the presence of both substances in the compound, however, based on these spectra, we cannot accurately calculate the DBP: AMP ratio and draw conclusions about the structure of the tracer.

Fluorescence spectra AMF and DBP-AMF were studied (Varian Cary Eclipse Fluorescence Spectrophotometer (American Laboratory Trading, Inc., USA). After excitation on 495 nm that emission for both substances (AMF and DBP-AMF) were observed on the same wavelength: 520 nm. This fact indicates that the conjugation of

AMP with the DBP derivative did not lead to a shift in the emission spectrum. Thus, to determine the tracer concentration, we can use the molar absorption coefficient for AMF 80000 M-1*cm-1 and concentration the DBP-AMF was 6 mkM.
https://www.aatbio.com/products/amf-4-aminomethyl-fluorescein-cas-91539-64-9#jump_overview

3. Binding curve fitting

The kinetic curves on Fig. 3 were approximated by exponential association equation (chosing the most appropruiate values of mP, mPmax and a):

$$mP = yo + a * (1 - \exp(-b * t)) \quad (1)$$

where mP – the varied FP signal (the y axis value), y0 – the FP signal of free DBP-AMF, t – time (the x axis value), a and b – are parameters termed the observed kinetics of the signal growth.

For curve 1 values y0, a and b are consists 53,4; 72.4 and 0.126, for curves 2 : 36.5; 48.7 and 0.076 and for curves 3 : 35, 64 and 2*10-5.

4. Detection of DBP from environmental water by GC-MS

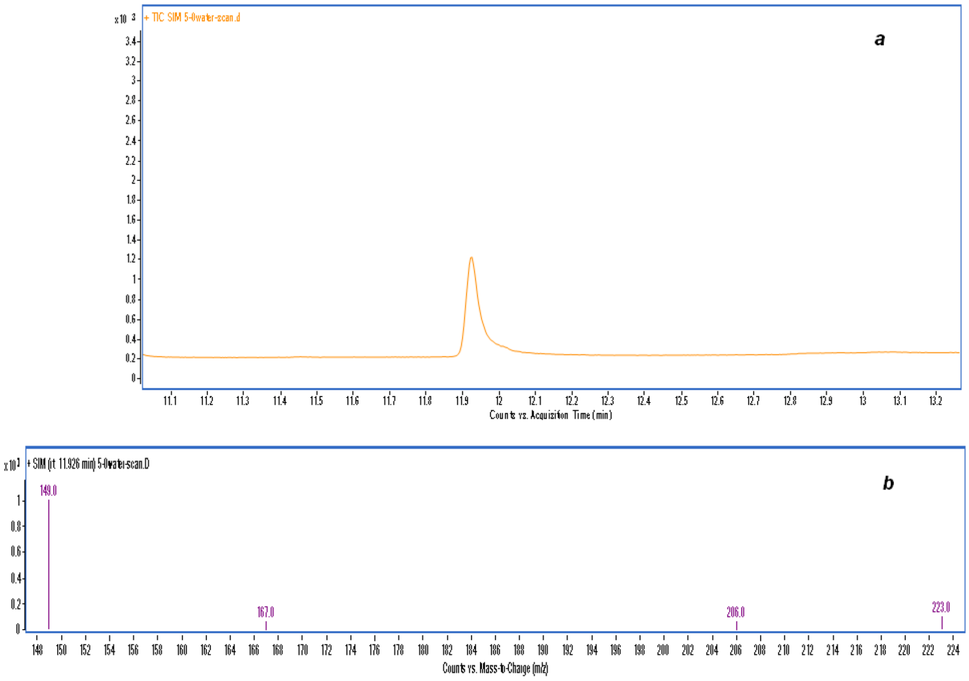

Figure S6. (a) Fragment of a chromatogram of a water extract containing DBP. (b) Experimental mass spectrum of the peak with a retention time of 11.93 min.

Figure S6a shows a fragment of the chromatogram of the aqueous extract containing DBP, which corresponds to the peak with a retention time of 11.93 min. The mass spectrum of this peak is shown in Figure S6b. A common fragment ion of protonated phthalate anhydride with a mass-to-charge ratio of m/z 149 was used to identify DBP. A minor peak of m/z 223 was used to confirm the detection of DBP.

5. Results of correlation both methods determination DBP in real water samples

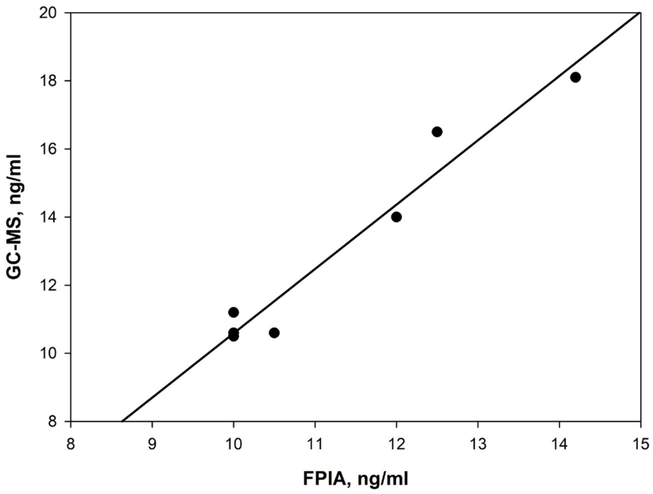

Figure S7. Correlation results determination DBP in real water samples by GC-MS and FPIA.

Disclaimer/Publisher's Note: The statements, opinions and data contained in all publications are solely those of the individual author(s) and contributor(s) and not of MDPI and/or the editor(s). MDPI and/or the editor(s) disclaim responsibility for any injury to people or property resulting from any ideas, methods, instructions or products referred to in the content.
